# *Aurelia aurita* Ephyrae Reshape a Coastal Microbial Community

**DOI:** 10.3389/fmicb.2016.00749

**Published:** 2016-05-19

**Authors:** Luca Zoccarato, Mauro Celussi, Alberto Pallavicini, Serena Fonda Umani

**Affiliations:** ^1^Marine Ecology Laboratory, Department of Life Science, University of TriesteTrieste, Italy; ^2^Oceanography Division, OGS (Istituto Nazionale di Oceanografia e di Geofisica Sperimentale)Trieste, Italy

**Keywords:** *Aurelia aurita*, ephyrae, microplankton, protists, prokaryotes, next-generation sequencing, community reshaping

## Abstract

Over the last two decades, increasing attention has been paid to the impact of jellyfish blooms on marine communities. *Aurelia aurita* is one of the most studied of the Scyphozoans, and several studies have been carried out to describe its role as a top-down controller within the classical food web. However, little data are available to define the effects of these jellyfish on microbial communities. The aims of this study were to describe the predation impact of *A. aurita* ephyrae on a natural microplanktonic assemblage, and to determine any reshaping effects on the prokaryote community composition and functioning. Surface coastal water was used to set up a 24-h grazing experiment in microcosms. Samples were collected to determine the variations in prey biomass, heterotrophic carbon production (HCP), extracellular leucine aminopeptidase activity, and grazing pressure. A next-generation sequencing technique was used to investigate biodiversity shifts within the prokaryote and protist communities through the small subunit rRNA tag approach. This study shows that *A. aurita* ephyrae were responsible for large decreases in the abundances of the more motile microplankton groups, such as tintinnids, Dinophyceae, and aloricate ciliates. Bacillariophyceae and Mediophyceae showed smaller reductions. No evidence of selective predation emerged in the analysis of the community diversity down to the family level. The heterotrophic prokaryote biomass increased significantly (by up to 45%), in parallel with increases in HCP and leucine aminopeptidase activity (40%). Significant modifications were detected in prokaryotic community composition. Some classes of Gammaproteobacteria and Flavobacteriia showed higher relative abundances when exposed to *A. aurita* ephyrae, while there was a net decrease for Alphaproteobacteria. Overall, this study provides new insight into the effects of *A. aurita* on microbial communities, underlining their selective predation toward the more motile groups of microplankton and their impact on prokaryotic assemblages, by favoring blooms of copiotrophic taxa.

## Introduction

Over the last two decades, jellyfish abundance and jellyfish blooms have become more frequent (Brotz et al., 2012). This has probably been related to human activities, such as overfishing and eutrophication, and to the increasing availability of new substrates (e.g., marine constructions) that are suitable for benthic stage settling (Richardson et al., 2009). Furthermore, several studies have highlighted that global warming is positively correlated to jellyfish abundance (Decker et al., 2007; Kogovšek et al., 2010; Lynam et al., 2011; Purcell, 2012). Some of the consequences that have arisen from these increased jellyfish numbers are well known, although not all of the ecological impacts have been unveiled. This study focused on the scyphomedusa *Aurelia aurita*, for which the trends for increasing bloom frequency (Kogovšek et al., 2010) and overall abundance (Mills, 2001) have been well documented. As *A. aurita* can adapt to a wide range of salinity and temperature, it is relatively common in the Adriatic Sea (Bonnet et al., 2012), where it forms dense aggregations, especially during spring and summer (Avian and Sandrini, 1994; Di Camillo et al., 2010).

*Aurelia aurita* has been largely studied as a top-down controller within the classical food web, with investigations into its ingestion of, or clearance rates for, rotifers, *Artemia salina*, mollusk larvae, fish larvae, copepods, and copepod nauplii (Båmstedt, 1990; Elliott and Leggett, 1997; Hansson et al., 2005; Titelman and Hansson, 2006; Møller and Riisgård, 2007; Riisgård and Madsen, 2011). The predation kinetics for these *A. aurita* prey are well known. *A. aurita* can consume up to 28,230 to 54,000 ind^-1^ d^-1^ (Omori et al., 1995), with ingestion rates that increase according to *A. aurita* size and seawater temperature (Båmstedt, 1990; Møller and Riisgård, 2007). Moreover the sizes of *A. aurita* and its prey have deep implications on the *A. aurita* capture efficiency (Riisgård and Madsen, 2011), although there are few studies in the literature that have tackled these issues to date.

One of the first studies that reported the presence of dinophyceae and ciliates in the *A. aurita* gut content was by Båmstedt (1990). Despite the low numbers detected for these prey (which were not a significant part of the *A. aurita* diet), and in agreement with data from Stoecker et al. (1987), Båmstedt (1990) proposed that different species have different vulnerabilities to *A. aurita* grazing. However, few studies have addressed the *A. aurita* feeding activity on microplanktonic organisms, as these studies have generally either focused on a few taxa (Uye and Shimauchi, 2005; Zheng et al., 2015) or on the microzooplankton community as a whole (Malej et al., 2007).

Jellyfish are also known to be an important source of dissolved organic matter (DOM) that can support the carbon demand of marine prokaryotes (Blanchet et al., 2014). The DOM that originates from jellyfish can integrate with or compensate for the DOM produced by phytoplankton (by primary production and exudation), especially in oligotrophic environments or during jellyfish outbreaks. This process has still been little investigated, and few data are available. Turk et al. (2008) indicated that a fraction of the released DOM is labile, as they detected significant shifts in terms of prokaryotic biomass and production during field experiments. The excretions of jellyfish contain inorganic nutrients (as mainly ammonium and phosphate) and DOM that is rich in primary amines and amino acids, which suggests tight coupling with prokaryotic activities, and hence an influence in carbon, nitrogen and phosphorus cycling (Pitt et al., 2009). Recently, Tinta et al. (2012) and Blanchet et al. (2014) demonstrated the bioavailability of jellyfish-derived organic matter (as homogenates of jellyfish bodies). Due to the protein-rich composition of this organic matter, rapid modifications of the prokaryote communities were triggered, which favored taxa that specialized in the degradation of complex compounds.

The aim of our study was thus to determine whether small jellyfish, as the ephyrae of *A. aurita*, can predate, and to determine their impact on the major microplankton groups within a natural coastal assemblage. Special effort was addressed to the description of the community compositions to a fine taxonomic resolution, to highlight possible selective *A. aurita* ingestion. We also aimed to describe the influence of *A. aurita* ephyrae on the composition and functioning of the prokaryote community.

## Materials and Methods

### *A. aurita* Ephyrae Collection and Seawater Sampling

During the last week of September 2014, *Crassostrea gigas* oysters bearing *Aurelia* polyps were collected by SCUBA diving from dock pillars in the Port of Koper (Slovenia). These were stored in containers filled with seawater that was collected at the sampling site, and transported to the laboratory at the Marine Biology Station (Piran, Slovenia). The polyps were kept in 0.45-μm-sieved seawater in the dark within a thermostatic chamber, and they were fed twice per week with freshly hatched *Artemia* nauplii *ad libitum*. The seawater was replaced 3 h after each feeding. The acclimation temperature was 19°C, and their asexual reproduction by transversal fissioning (‘strobilation’) was induced by lowering the temperature to 14°C. The ephyrae were then fed with freshly collected zooplankton (50-μm net) until 36 h before the setup of the experiment to limit contamination with eukaryotic DNA from the medium. Once the ephyrae reached the desired mean size of 5 mm in diameter (October 27, 2014), they were transferred as quickly as possible to the Laboratory of Marine Ecology (University of Trieste, Italy). The seawater for the experiments was freshly collected from the surface waters in Aurisina Bay (Trieste, Italy), a few meters from the coastline, and it was immediately filtered through 200-μm mesh to remove any organisms larger than microplankton.

### Ephyrae – Grazing Experiment

The filtered seawater was immediately transferred to the laboratory and used to prepare six microcosms in 2.2-L transparent bottles (Nalgene): three bottles were used as controls, and three bottles were used as treatments, where five ephyrae were added to each. The microcosms were then placed into an aquarium with flowing water, and incubated for 24 h, exposed at *in-situ* photosynthetically active radiation (26 μmol m^-2^ s^-1^) and temperature (16.8–17.5°C); irradiance followed the natural day–night cycle. To avoid sedimentation, the bottles were gently inverted every hour.

The impact of the ephyrae on natural microbial communities was assessed according to several classes of microbes: pigmented and heterotrophic nanoplankton, and autotrophic and heterotrophic prokaryotes. The samples for analysis were taken at the beginning (T0) and at the end (T24) of the incubation, for determination of the following: abundance and biomass of each microbe class, leucine aminopeptidase exoenzymatic activity, heterotrophic carbon production (HCP), and diversity of microplankton and prokaryotes using next-generation sequencing (NGS). At T0, the samples were taken directly from the filtered seawater as three replicates, while at T24, the samples were taken from each microcosm. During the incubation, further samples were taken for estimation of nanoplankton and prokaryotic abundance, leucine aminopeptidase activity, and HCP. According to the protocol of Frost (1972), the abundances and biomasses of microplanktonic taxa at T0 and T24 were used to estimate their growth and grazing coefficients, and the mean growth and grazing coefficients from each ephyra-treated microcosm were used to calculate the ephyra ingestion rates. The abundances and biomasses of microplankton were estimated to the finest taxonomic level reachable by the operators at the optical microscope (i.e., family, genus, species). When a taxon was missing at T24 within an ephyrae-treated microcosm, the arbitrary value of 1 was given, to allow the formula calculations. Only ingestion rates greater than twofold their own standard deviation were considered.

### Microscopic Analysis for Abundance and Biomass

#### Microplankton

For each sample, an aliquot of 0.5 L seawater was fixed with 2% buffered formaldehyde solution (final concentration), and stored in a dark bottle at 4°C. The whole volume was processed following Utermöhl (1958). Using an inverted optical microscope (Olympus IX51), the organisms were assigned taxonomically, and enumerated and measured with an eye piece. The geometrical formulae summarized in Olenina et al. (2006) were used to estimate the biovolumes of the Dinophyceae, Coccolithophyceae, Coscinodiscophyceae, Fragilariophyceae, Dictyochophyceae, Mediophyceae, and Bacillariophyceae. For the aloricate ciliates, tintinnids, and metazoans, the biovolumes were calculated from the equivalent geometrical shapes (Edler, 1979). The equations from Menden-Deuer and Lessard (2000) were then used to obtain the organic carbon quotas.

#### Nanoplankton and Prokaryotes

Samples of 20 and 3 mL were taken to determine the abundances of nanoplankton and prokaryotes, respectively. The samples were fixed with 2% buffered formaldehyde solution (final concentration; prefiltered through 0.2-μm Acrodisc syringe filters), stored in sterile dark bottles at 4°C, and processed following Porter and Feig (1980). Each sample of prokaryotes was processed as three replicates. Aliquots of each sample were stained with 1 μg mL^-1^ 4′,6-diamidino-2-phenylindole (DAPI; final concentration) and placed in the dark for 15 min. After staining, prokaryotes were collected on 0.22-μm black polycarbonate filters (diameter, 25 mm; Nucleopore), while nanoplankton was collected on 0.8-μm black polycarbonate filters (diameter, 25 mm; Nucleopore). The filters were then immediately placed on slides between two drops of non-fluorescent immersion oil (Olympus), and kept at -20°C in the dark.

The counting was carried out using an epifluorescence microscope (Olympus BX 60 F5) at a final magnification of 1000×, with a UV filter set for DAPI (BP 330–385 nm; BA 420 nm), and green (BP, 480–550 nm; BA, 590 nm) and blue (BP, 420–480 nm; BA, 515 nm) light sets for natural pigment fluorescence. More than 300 cells were counted for prokaryotes and nanoplankton in each sample; non-pigmented cells were considered as heterotrophic.

For the biomass estimation of nanoplankton, these were divided into three classes according to their dimensions: 2–3 μm, 3–5 μm, and 5–10 μm, as reported by Christaki et al. (2001). The cell abundances were converted in biomass by applying the following conversion factors: 20 fg C cell^-1^ for heterotrophic prokaryotes (Ducklow and Carlson, 1992) and 200 fg C cell^-1^ for *Synechococcus* (Caron et al., 1991). Nanoplanktonic organisms were approximated to spheres (diameter as the mean of each dimensional class), to multiply their volumes by the conversion factor of 183 fg C μm^-3^ (Caron et al., 1995).

### Heterotrophic Carbon Production

Heterotrophic carbon production was estimated by incorporation of [^3^H]-leucine (Kirchman et al., 1985). At each sampling time, duplicate 1.7-mL aliquots and one ‘killed’ control (by addition of 90 μL 100% trichloroacetic acid) were collected from each microcosm, and 20 nM of the radiotracer (specific activity, 52.9 Ci mmol^-1^) was added, followed by incubation at 16.8–17.5°C in the dark. These incubations were stopped after 1 h by the addition of 5% trichloroacetic acid (final concentration). Extraction with 5% trichloroacetic acid and 80% ethanol was then carried out using the microcentrifugation method (Smith and Azam, 1992). The radioactivity of the [^3^H]-leucine incorporated into the samples was determined using a β-scintillation counter (Tri-Carb 2900 TR Liquid Scintillation Analyzer, Packard) after the addition of 1 mL scintillation cocktail (Ultima Gold MV; Packard). This incorporation of [^3^H]-leucine was converted into carbon produced via prokaryotic protein production, according to Simon and Azam (1989), assuming twofold isotope dilution for leucine. The mean coefficient of variation among the replicates was 3%.

### Leucine Aminopeptidase Activity

Leucine aminopeptidase activity was determined using the fluorogenic substrate analog (Hoppe, 1993) leucine-7-amino-4-methyl-coumarin (Sigma–Aldrich). The enzyme activity was expressed in terms of the rate of 7-amino-4-methyl-coumarin (AMC) production over time. This hydrolysis reaction was measured by incubation of triplicate 2-mL sub-samples collected at each time point from each of the microcosms with 200 μM substrate (saturating concentration; Celussi and Del Negro, 2012) for 1 h in the dark at the experimental temperature. Fluorescence increases due to AMC production from the substrate were measured using a spectrofluorometer (excitation, 380 nm; emission, 440 nm; Shimadzu RF-1501). Triplicate standard AMC solutions (Sigma–Aldrich) were used to construct the calibration curves. Duplicate blanks without the fluorogenic substrate were used to determine the natural fluorescence increase in the samples that was not attributable to the enzyme activity. To determine the degradation processes performed by the prokaryotic consortium associated to the ephyrae, at T24, three ephyrae were collected alive and placed one each in three vials with 1 mL seawater from the same bottle. The vials had 9 mL 0.2-μm-filtered seawater added, for a 1:10 dilution. To estimate the leucine aminopeptidase activity, these aliquots were treated as described above, with 200 μM final substrate concentration. The substrate hydrolysis ascribable to ephyra-associated prokaryotes were then computed by correcting the data according to the dilution (i.e., 10x) and subtracting the hydrolysis rates measured in the ephyra-free seawater.

### Next-Generation Sequencing: Sample Collection and Processing

The molecular diversity descriptions were based on metabarcoding analysis performed with an Ion Torrent personal genome machine (PGM) platform. For the microplankton community, the hyper variable region 9 of the rRNA 18S gene was targeted with the primer pair of 1391F and EukB (Stoeck et al., 2010). Samples were collected, with 1 L filtered per sample on 2-μm PCTE membranes (Sterlitech), and the membranes were immediately frozen at -80°C.

Extraction of the DNA was performed using PowerSoil DNA Isolation kits (Mobio), with a customized protocol that included two improvements: the membranes were completely dissolved in chloroform during the DNA extraction step, to increase the DNA recovery so as to avoid issues related to the folding and scrubbing of the filter; and the chloroform was then removed through the increase of the first centrifugation step to 5 min, and the recovery of the upper aqueous phase. To limit over-cycling, amplification of the targeted region was followed in real time, and the amplification of each sample was stopped when the plateau was reached. The primary qPCR was performed in 10-μL reactions with 0.5 U KAPA 2G HiFi Taq, 1x KAPA 2G Buffer HiFi, 0.3 μM dNTPs, 1x EvaGreen (Biotium), 0.3 μM of each primer, 2 μL DNA template, and RNase-free water to the final volume. The thermocycling conditions were set to: 1 min at 95°C, 28–31 cycles of 15 s at 95°C, 10 s at 60°C, 4 s at 72°C, and 3 min final elongation at 72°C. Negative controls with RNase-free water instead of DNA template were amplified to ensure the absence of contamination. Sequencing adapters were attached to the amplicons, with a secondary PCR performed in 25 μL with the same reagents, concentrations and cycling conditions as the primary PCR (nine cycles). There was no significant amplification seen in the negative controls.

For the prokaryotic community, the hypervariable region 4 of the rRNA 16S gene was targeted. The samples were collected by filtering 0.5 L for each sample with 0.2-μm cellulose acetate membranes (Sterlitech) to collect the organisms, and the membranes were then immediately frozen at -80°C.

Extraction of the DNA was performed with PowerSoil DNA Isolation kits (Mobio) following the original protocol. The PCR amplification strategy was the same as that used for microplankton. The primers for the primary qPCR were 515F (S-*-Univ-0515-a-S-19) and a combination of 806R (S-D-Bact-0787-b-A-20) with 802R (S-D-Bact-0785-b-A-18; Claesson et al., 2010; Walters et al., 2011). The PCR reactions were performed in 10 μL with 1x HotMasterMix (5 PRIME), 1x EvaGreen (Biotium), 0.3 μM forward primer, and 0.15 μM and 0.15 μM reverse primers, 2 μL DNA template, and RNase-free water to the final volume. The thermocycler conditions were set to: 2 min at 94°C, 26–34 cycles of 20 s at 94°C, 20 s at 55°C, 40 s at 65°C, and 2 min final elongation at 65°C. The secondary qPCR was performed in 25 μL with the same reagents, concentrations and cycling conditions as for the primary PCR (nine cycles). The dilution of the DNA template and the primer pair for the secondary PCR were the same as for the microplankton secondary PCR.

The amplified samples were normalized using SequalPrep Normalization kits (Thermo Fisher), pooled together, and processed with Ion PGM Hi-Q OT2 kits and Ion PGM Hi-Q Sequencing kits (Life Technologies). The sequencing was carried out with an Ion Torrent PGM running Ion 314 chip v2 for microplankton samples and Ion 316 chip v2 for prokaryote samples.

### Bioinformatics Analysis

The read dataset was exported raw from Torrent Server in fastq format. Demultiplex and forward primer removal was carried out with fastq_strip_barcode_relabel2.py script (USEARCH package^1^), while the reverse primers and reverse adaptors were trimmed with cutadapt 1.8.3 (Martin, 2011). The mean quality scores of the reads were checked with FastQC; the length and quality filtration were carried out with USEARCH v8 (Edgar and Flyvbjerg, 2015), setting the minimum length threshold at 70 b and 150 b for the microplankton and prokaryote sequences, respectively, and the quality threshold to a maximum that allowed expected errors of one nucleotide per 100 bases. All of the sequences have been deposited in GenBank (NCBI), and the BiomProject accession numbers for the 18S dataset is PRJNA305513, and for the 16S dataset is PRJNA305512. Chimeras were removed using the UCHIME algorithm (Edgar et al., 2011), whereby *de novo* chimera detection was chosen for the microplankton dataset, and the prokaryote dataset was screened using the GreenGenes v13.8, with the representative dataset clustered at 97% similarity. Operational taxonomic unit (OTU) picks for both cleaned sequence datasets were carried out in QIIME, using the open reference workflow strategy, where singletons were removed and the taxonomic assignment of the OTUs was performed with BLAST (Altschul et al., 1990), setting the *e*-value > 10^-20^. The PR2 reference dataset was used as the reference for sequences of microplankton, while the GreenGenes v13.8 reference dataset (clustered at 97% similarity) was chosen for the prokaryote dataset.

### Statistical Analysis

The ingestion rates (cells ind^-1^ d^-1^) estimated for each ephyra treatment were standardized as relative ingestion rates (%) by dividing the ingestion of each taxon by the sum of the ingestion of all of the taxa. For all of the analytical replicates at T0, the relative initial abundances (%) of each taxa was obtained by dividing by the total abundance of microplankton (cells L^-1^). The correlation between the relative ingestion rates and the relative initial abundances, and the biovolumes, were tested using the Pearson (P) and Spearman (S) indices.

The microplankton OTU table was manually cleaned from multicellular organisms such as Archaeplastida, Metazoa, Amoebozoa, and Fungi, as the sampling method was not representative for these groups. Therefore only protists were considered further. A multiple rarefaction step was applied to both protist and prokaryote tables to minimize differences due to sequencing depth. Similarity matrices were calculated and constructed using the Bray–Curtis similarity coefficient. Hierarchical cluster analysis (clustering on the group means) was achieved using Primer 6 (PRIMER-E Ltd., Plymouth, UK).

The variations in the community compositions were tested for significance in R^2^, with analysis of similarity (ANOSIM) using *a priori*–defined groups (T0, T24; controls, treatments) and the groups that were highlighted through the cluster analysis. The R_ANOSIM_ statistical values that were generated by ANOSIM are the relative measures of separation of the *a priori*–defined groups. A zero (0) indicates that there was no difference among the groups, while a one (1) indicates that all of the samples within the groups were more similar to one another than any of the samples from the different groups.

To compare the taxa composition among the samples, the relative abundances (RAs) were calculated from the OTU abundance of both protists and prokaryotes. Only OTUs in all three replicates of the T0 and T24 controls, and the T24 ephyra treatments, were kept, and OTUs with <1% RA were combined as the category of ‘Others.’ The RA community profiles were plotted using Microsoft Excel. The *DESeq* package in R was used to test for significant variations in the taxa RAs between T0 and T24.

## Results

The natural assemblage of microbes at T0 in this experiment was dominated by prokaryotes, which constituted >80% of the total biomass. Of this, heterotrophic prokaryotes represented 12.19 ± 1.13 μg C L^-1^, and *Synechococcus* represented 5.37 ± 0.02 μg C L^-1^. The nanoplankton biomass (as both heterotrophic and phototrophic fractions) was 0.65 ± 0.06 μg C L^-1^, and the microplankton biomass was 3.60 ± 0.58 μg C L^-1^. After the 24-h incubation (i.e., at T24), two different scenarios occurred. In the controls, *Synechococcus* increased, to reach the biomass of 7.59 ± 0.61 μg C L^-1^, while all of the other group showed almost constant biomasses from T0. However, in the presence of the ephyrae, the heterotrophic prokaryote biomass increased to 17.60 ± 0.70 μg C L^-1^, whereas for microplankton, there was a large decrease to 1.49 ± 0.26 μg C L^-1^.

**FIGURE 1:**
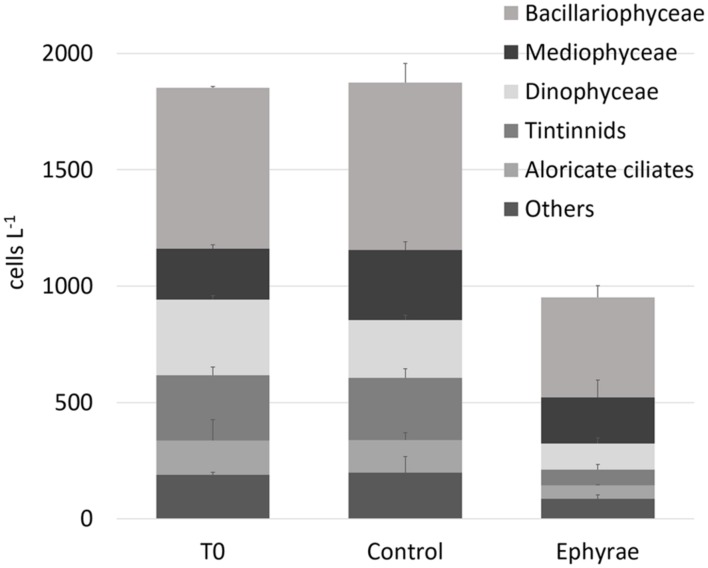
**Abundances (±SD) of major microplanktonic groups at T0 and T24 for the control and ephyra-treatment microcosms.** The taxa Dictyochophyceae, Metazoa, Coccolithophyceae, Coscinodiscophyceae, and Fragilariophyceae are grouped together as “Others.”

**Figure 1** illustrates the overall abundances of the major groups that were detected in the microplankton community at T0 and T24, for all of the six microcosms. The natural assemblage (T0) was mainly composed of 40% Bacillariophyceae, 17% Mediophyceae, 14% Dinophyceaea, 12% tintinnids, and 7% aloricate ciliates. Dictyochophyceae, Metazoa, Coccolithophyceae, Coscinodiscophyceae, and Fragilariophyceae were grouped within the category of ‘Others,’ with a relative abundance of <4%. No significant variations were detected for these total abundances between the T0 and T24 controls (Mann–Whitney test, *p* = 0.31), and also not in the group composition (ANOSIM, *R* = 0.25, *p* = 0.4). The comparison between T0 and the T24 ephyra treatments also showed no significant variations (ANOSIM, *R* = 1, *p* = 0.1), although decreased abundance was detected for tintinnids (–76%), Dinophyceaea (–65%), and aloricate ciliates (–61%), while Bacillariophyceae and Mediophyceae showed smaller decreases (-38, -10%, respectively).

Within the aloricate ciliates and tintinnids, the organisms belonging to the Ciliophora, Strombidiidae, and *Tintinnopsis* sp. taxa showed broad variability in size during the microscopy counting, and these were thus split into different size ranges (**Table 1**). The mean ingestion rates of a single *A. aurita* ephyra during the grazing experiment (**Table 1**) generally showed that the most affected groups were Bacillariophyceae and tintinnids. These included several taxa that were strongly preyed upon by the *A. aurita* ephyrae, such as: undetermined Bacillariophyceae, with an ingestion rate of 34.5 ± 11.3 cells ind^-1^ d^-1^ (corresponding to 0.15 ± 0.05 × 10 μg C ind^-1^ d^-1^); *Pseudonitzschia* sp., with an ingestion rate of 36.4 ± 3.8 cells ind^-1^ d^-1^ (0.06 ± 0.01 × 10 μg C ind^-1^ d^-1^); and *Stenosemella nivalis*, with an ingestion rate of 38.2 ± 2.4 cells ind^-1^ d^-1^ (0.65 ± 0.04 × 10 μg C ind^-1^ d^-1^). High ingestion rates were found for some taxa of the other groups, such as: the Dinophyceae *Ceratium kofoidii*, with an ingestion rate of 20.2 ± 5.7 cells ind^-1^ d^-1^ (0.31 ± 0.09 × 10 μg C ind^-1^ d^-1^); the aloricate ciliate Strombidiidae C2 (the sub-group of Strombidiidae characterized by larger size), with an ingestion rate of 12.1 ± 0.8 cells ind^-1^ d^-1^ (0.21 ± 0.01 × 10 μg C ind^-1^ d^-1^); the Mediophyceae *Leptocylindrus* sp. with an ingestion rate of 42.8 ± 0.5 cells ind^-1^ d^-1^ (0.18 ± 0.00 × 10 μg C ind^-1^ d^-1^); and the only Coscinodiscophyceae *Guinardia striata*, with an ingestion rate of 28.1 ± 0.0 cells ind^-1^ d^-1^ (0.03 ± 0.00 × 10 μg C ind^-1^ d^-1^).

**Table 1:** Biovolume, initial (T0) cell abundance/biomass (±SD computed on analytical replicates) and final (T24) *A. aurita* ephyra ingestion rate calculated on abundance/biomass (±SD computed on experimental replicates) for each preyed taxa of microplankton.

Taxa	Biovolume (μm^3^)	T0		T24: Ingestion rates	
			
		Abundance (cells L^-^^1^)	Biomass (×10 μg C L^-^^1^)	(cells ind^-^^1^ d^-^^1^)	(×10 μg C ind^-^^1^ d^-^^1^)
**Dinophyceae**
*Ceratium furca*	73236	31.5 ± 7.8	2.52 ± 0.62	9.9 ± 1.0	0.79 ± 0.08
*Ceratium kofoidii*	12747	151 ± 14.1	2.34 ± 0.22	20.2 ± 5.7	0.31 ± 0.09
*Dinophysis fortii*	80953	5.0 ± 1.4	0.44 ± 0.12	1.0 ± 0.0	0.06 ± 0.05
*Gonyaulax* sp.	134628	17.5 ± 4.9	2.48 ± 0.70	4.2 ± 0.8	0.59 ± 0.11
*Oxytoxum caudatum*	8177	3.5 ± 0.7	0.04 ± 0.01	0.9 ± 0.3	0.01 ± 0.01
*Prorocentrum micans*	43960	19 ± 9.9	0.94 ± 0.49	4.7 ± 1.7	0.23 ± 0.08
Dinophyceae undet.	58875	4.0 ± 2.8	0.26 ± 0.18	2.5 ± 0.4	0.16 ± 0.03
**Aloricate ciliates**
Ciliophora (A1)^a^	14137	34.5 ± 13.4	0.59 ± 0.23	5.0 ± 0.5	0.09 ± 0.01
Ciliophora (A2)^a^	65450	10.5 ± 2.1	0.75 ± 0.15	2.5 ± 0.9	0.18 ± 0.06
Strombidiidae (C1)^a^	3142	20 ± 4.2	0.08 ± 0.02	8.2 ± 0.7	0.03 ± 0.00
Strombidiidae (C2)^a^	14544	31 ± 9.9	0.54 ± 0.17	12.1 ± 0.8	0.21 ± 0.01
**Tintinnids**
*Favella* sp.	13901	60.5 ± 10.6	1.02 ± 0.18	13.8 ± 3.0	0.23 ± 0.05
*Stenosemella nivalis*	14137	101.5 ± 21.9	1.73 ± 0.37	38.2 ± 2.4	0.65 ± 0.04
*Tintinnopsis* sp. (E1)^a^	4712	49.5 ± 9.2	0.30 ± 0.06	14.6 ± 2.5	0.09 ± 0.02
*Tintinnopsis* sp. (E2)^a^	21817	19.5 ± 12.0	0.50 ± 0.31	7.9 ± 1.2	0.20 ± 0.03
*Tintinnopsis* sp. (E3)^a^	59865	3.5 ± 2.1	0.23 ± 0.14	1.9 ± 0.1	0.13 ± 0.01
**Metazoa**
Copepod nauplii	176625	10.0 ± 0.0	1.83 ± 0.00	2.2 ± 0.5	0.27 ± 0.24
**Coccolithophyceae**
*Calciosolenia murray*	4091	50.5 ± 38.9	0.12 ± 0.10	5.7 ± 0.4	0.01 ± 0.00
*Cocconeis* sp.	2322	38.5 ± 14.8	0.06 ± 0.02	6.6 ± 2.1	0.01 ± 0.00
**Bacillariophyceae**
*Diploneis* sp.	7800	29.0 ± 24.0	0.12 ± 0.10	7.7 ± 0.4	0.03 ± 0.00
*Navicula* sp.	4377	85.0 ± 7.1	0.22 ± 0.02	15.0 ± 0.5	0.04 ± 0.00
Bacillariophyceae undet.	8450	248 ± 31.1	1.09 ± 0.14	34.5 ± 11.3	0.15 ± 0.05
*Pseudonitzschia* sp.	2500	142 ± 39.6	0.23 ± 0.06	36.4 ± 3.8	0.06 ± 0.01
*Pleurosigma* sp.	10838	126 ± 31.1	0.68 ± 0.17	23.2 ± 7.9	0.12 ± 0.04
**Mediophyceae**
*Chaetoceros* sp.	4352	11.5 ± 2.1	0.03 ± 0.01	4.4 ± 1.6	0.01 ± 0.00
*Leptocylindrus* sp.	7850	167 ± 72.1	0.69 ± 0.30	42.8 ± 0.5	0.18 ± 0.00
**Fragilariophyceae**
*Fragilaria* sp.	1313	59.0 ± 38.2	0.06 ± 0.04	28.1 ± 0.0	0.03 ± 0.00
**Coscinodiscophyceae**
*Guinardia striata*	49063	11.5 ± 14.8	0.21 ± 0.27	3.9 ± 0.3	0.07 ± 0.01


*^a^1 to 3, increasing size groups; A, sphere; C, cone; E, cylinder; as geometric shapes used to compute biovolumes.*

**FIGURE 2:**
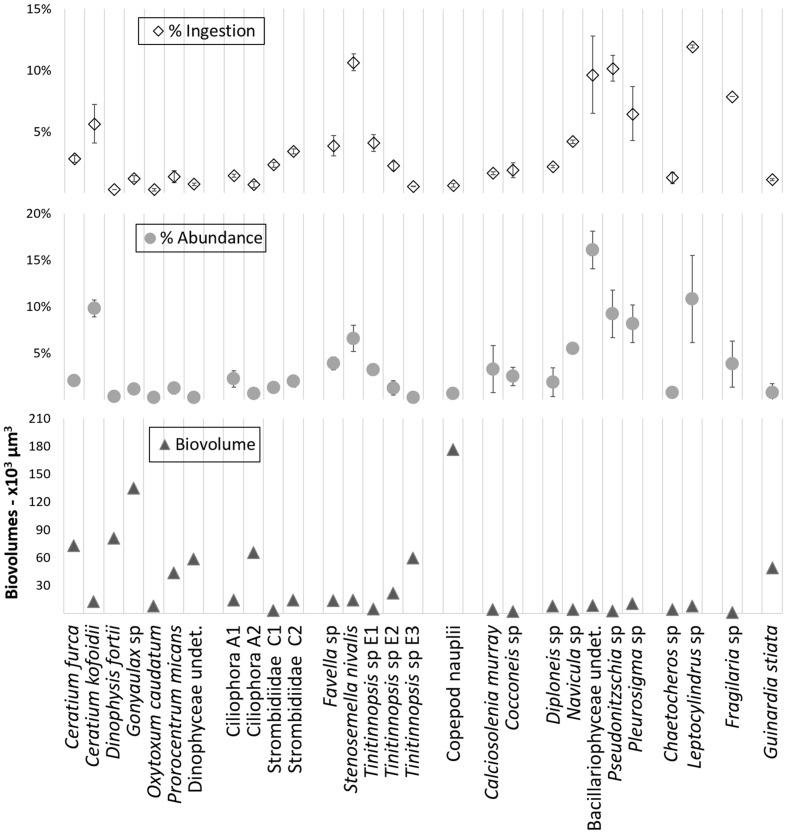
**Overview of the relative ingestion rates, relative initial abundances, and biovolumes determined with the grazing experiment for microplanktonic taxa**.

The relative ingestion rates also paralleled the relative initial abundances of the taxa that were preyed upon by the *A. aurita* ephyrae (**Figure 2**), with significant linear (*P* = 0.87, *p* < 0.001) and rank-order (*S* = 0.92, *p* = < 0.001) correlations, Conversely, there was no significant correspondence between the *A. aurita* ephyra ingestion and the prey biovolumes. *Ceratium kofoidii*, *Stenosemella nivalis*, undetermined Bacillariophyceae, *Pseudonitzschia* sp., *Pleurosigma* sp., and *Leptocylindrus* sp. were the most abundant taxa, and these also showed the highest ingestion, although their biovolumes were among the smallest, as these were each <15 × 10^3^ μm^3^. Taxa that were characterized by higher biovolumes were among the least ingested, such as *Ceratium furca*, *Dinophysis fortii*, *Gonyaulax* sp., *Ciliophora* A2, *Tintinnopsis* sp. E3, and Copepod nauplii (from 60 × 10^3^ μm^3^ to 177 × 10^3^ μm^3^).

**FIGURE 3:**
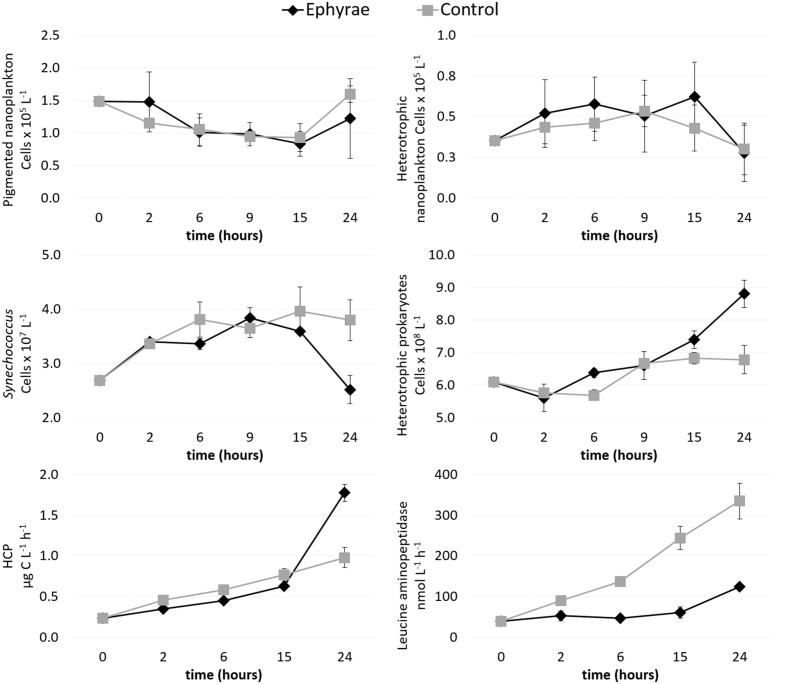
**Time courses of pigmented nanoflagellate abundance, heterotrophic nanoflagellate abundance, *Synechococcus* abundance, heterotrophic prokaryote abundance, heterotrophic carbon production (HCP), and leucine aminopeptidase activity.** Data are means (±SD) (*n* = 3) for the control (gray) and ephyra-treatment (black) microcosms.

The heterotrophic and pigmented nanoplankton abundances remained almost constant over the incubation period, both in the control microcosms and in those with the ephyrae (**Figure 3**). Within the first 9 h of incubation, *Synechococcus* numbers showed an increasing trend in both the control and ephyrae microcosms. Their abundances at the end of the incubations (T24) saw the controls increase as the number of cells, to 2.22 ± 0.76 cells × 10^7^ L^-1^, while with the ephyra treatment, the numbers decreased back to the initial (T0) values. The abundance of heterotrophic prokaryotes similarly increased within the first 9 h in both series of microcosms, while at T24, in the controls the number of cells was slightly higher than at T0, and with the ephyra treatment, this was significantly increased to 5.42 ± 0.85 cells × 10^7^ L^-1^ (*F* = 31.65, *p* < 0.01).

Heterotrophic carbon production increased over time in all of the microcosms, with the lowest values measured at T0 (0.23 ± 0.01 μg C L^-1^ h^-1^). The first 15 h of the incubations were characterized by moderate increases in the C uptake, with the controls (as mean ± SD of all replicates: 0.62 ± 0.01 μg C L^-1^ h^-1^) slightly lower than in the presence of the ephyrae (0.77 ± 0.08 μgC L^-1^ h^-1^). By T24, the highest HCP was in the controls (1.78 ± 0.11 μg C L^-1^ h^-1^), whereas with the ephyrae this remained almost half the control (0.98 ± 0.12 μg C L^-1^ h^-1^).

Leucine aminopeptidase activity increased linearly over time in the ephyra-treated microcosms, from 39.01 ± 0.76 nM h^-1^ (as mean ± SD of all replicates) at T0, to 334.56 ± 44.42 nM h^-1^ at T24. On the contrary, polypeptide degradation in the controls remained relatively constant over the first 15 h (49.61 ± 11.33 nM h^-1^), with a small increase at T24 (132.52 ± 0.32 nM h^-1^). At T24, the leucine aminopeptidase activity of the prokaryotic consortium associated to the ephyrae was 1083.47 ± 60.90 nM h^-1^ per individual.

**FIGURE 4:**
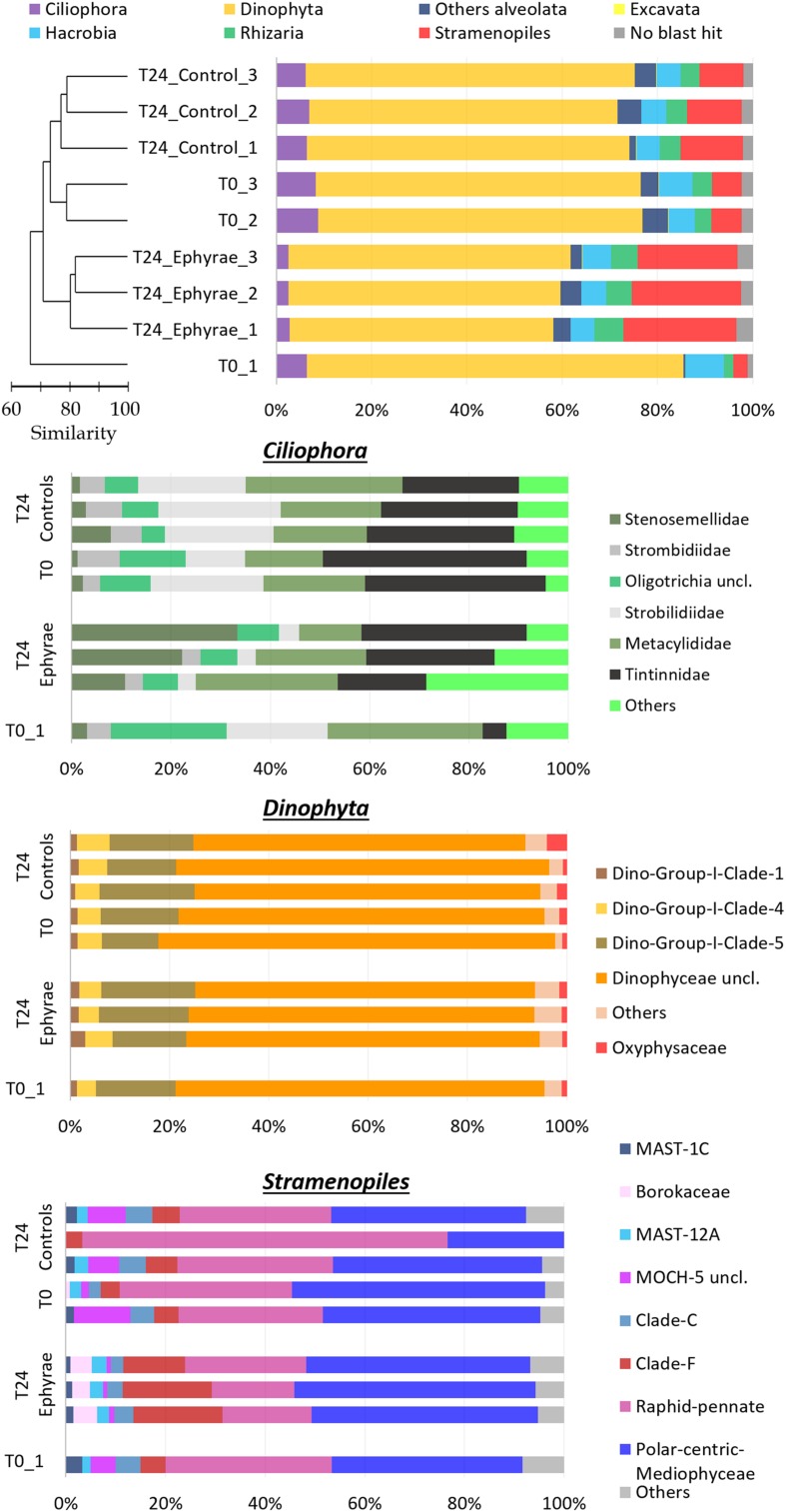
**Profiles of the protist community composition obtained with the NGS technique.** **(Top)** The major phyla (major division for phylum Alveolata), with the profiles ordered according to the cluster analysis shown next to the *Y*-axis. **(Bottom)** Three bar plots showing the family compositions of Ciliophora, Dinophyta, and Stramenopiles. Taxa with RAs < 1% were grouped as “Others” (RA < 5% for Ciliophora).

The effects of this ephyra predation on the protist community were investigated by analyzing the sequences obtained through parallel mass sequencing techniques (**Figure 4** and **Supplementary Figure 1**; Top). Compared to the microplankton community analyzed under the microscope, this protist community was lacking in Metazoa, which had an abundance of only 1%. In the natural assemblages (i.e., at T0), the phylum of Alveolata was the most abundant, with RAs from 80.2 to 85.8%. These Alveolata were mainly composed of the divisions Dinophyta (>80%) and Ciliophora (>7%). The other phyla detected were Stramenopiles (RA, 3.0 to 23.7%), Hacrobia (RA, 4.9 to 8.0%), and Rhizaria (RA, 2.0 to 4.2%), with low presence of Excavate (RA, ∼0.1%). Within the phylum Alveolata, there were also sequences belonging to the division Apicomplexa, as well as Choanoflagellates being detected within the phylum Opisthokonta. These taxa, plus the other unclassified sequences, were grouped together in the category of ‘other Alveolata’ because of their low RAs. The sequences that did not show any hits during the BLAST analysis and that remained unclassified were always <3.4% of the total sequences among all of the samples.

This NGS-based protist diversity analysis was particularly productive compared with the microscopy analysis. For the most represented phylum, the identification included 29 families of Stramenopiles, 15 families of Ciliophoran, and 21 families of Dinophyta, while the taxa of Excavate and Rhizaria (at 2 and 16 families, respectively) were completely missing from the microscopy analysis. The cluster analysis for the community profiles of all of the samples showed that replicates 2 and 3 at T0 and all of the replicates of the T24 controls grouped together with 73% similarity (this cluster will henceforth be referred to as ‘EUK_1’). Similarly, the replicates of the T24 ephyra treatments grouped at 80% similarity (henceforth referred to as ‘EUK_2’). T0 replicate 1 showed a peculiar assemblage that was mostly unrelated to the other replicates.

Comparing the two clusters (EUK_1 and EUK_2) with ANOSIM, a significant difference emerged (*R* = 0.81, *p* < 0.05). The mean RA of Dinophyta for EUK _1 was 67.6 ± 1.7%, which was significantly higher than that for EUK_2, at 57.3 ± 2.0% (Mann–Whitney test, *p* < 0.05). Also, the mean RA of Ciliophora was significantly higher for EUK_1 (7.3 ± 1.2%) than EUK_2 (2.63 ± 0.2%; Mann–Whitney test, *p* < 0.05). On the other hand, the Stramenopiles RA for EUK_1 (9.3 ± 3.1%) was significantly lower than EUK_2 (22.5 ± 1.5%; Mann–Whitney test, *p* < 0.05). For the other taxa, such as Hacrobia, Rhizaria and the category of ‘other Alveolata,’ these showed variations among the clusters that were comparable to the variation within each cluster.

Insight analysis was carried out for the taxa composition at the family level for the phyla with the highest variations between EUK_1 and EUK_2 (i.e., Ciliophora, Dinophyta, Stramenopiles; **Figure 4** and **Supplementary Figure 1**; Bottom), with RAs of these taxa recomputed for each phylum. Ciliophora showed the highest number of abundant taxa, with six families with RA > 5%, and 13 families with RA > 1%. Combined with a possible issue arising from sampling depth – as the RA of Ciliophora within the protist community was very low – this complexity might lead to the fuzzy variation in the RAs of these taxa. However, comparing EUK_1 and EUK_2, Strombilidiidae showed a significant net decrease in RA (*DESeq* test, *p* < 0.001). Also, Tintinnidae showed a decrease, although less evident, while Stenosemellidae showed increased RA (*DESeq* test, both *p* < 0.001). Despite the overall decrease in the RA from EUK_1 to EUK_2, Dinophyta did not show any particular changes in the RAs among its families (*DESeq* test, all *p* > 0.05). The Dinophyceae unclassified taxon was the most abundant among all of these profiles (RA, 47.2 ± 6.7% among all protists; RA, 72.1 ± 4.0% among Dinophyta taxa), so further insight analysis was carried out at the genus level to determine this composition. However, from 58.3 to 73.4% of the reads for this taxon still remained unclassified, while among the more abundant genera there were *Gyrodinium*, *Gonyaulax*, *Gymnodinium*, *Pentapharsodinium*, *Alexandrium*, and *Prorocentrum* (RAs, 2.5–5.9%). Stramenopiles showed variable RAs for some of the families in EUK_1 (especially for the community profile of T24 control replicate 2). Here, comparing EUK_1 and EUK_2, the RAs of the Raphid–Pennate decreased, while clade-F (class Chrysophyceae–Synurophyceae) increased (*DESeq* test, both *p* < 0.001).

**FIGURE 5:**
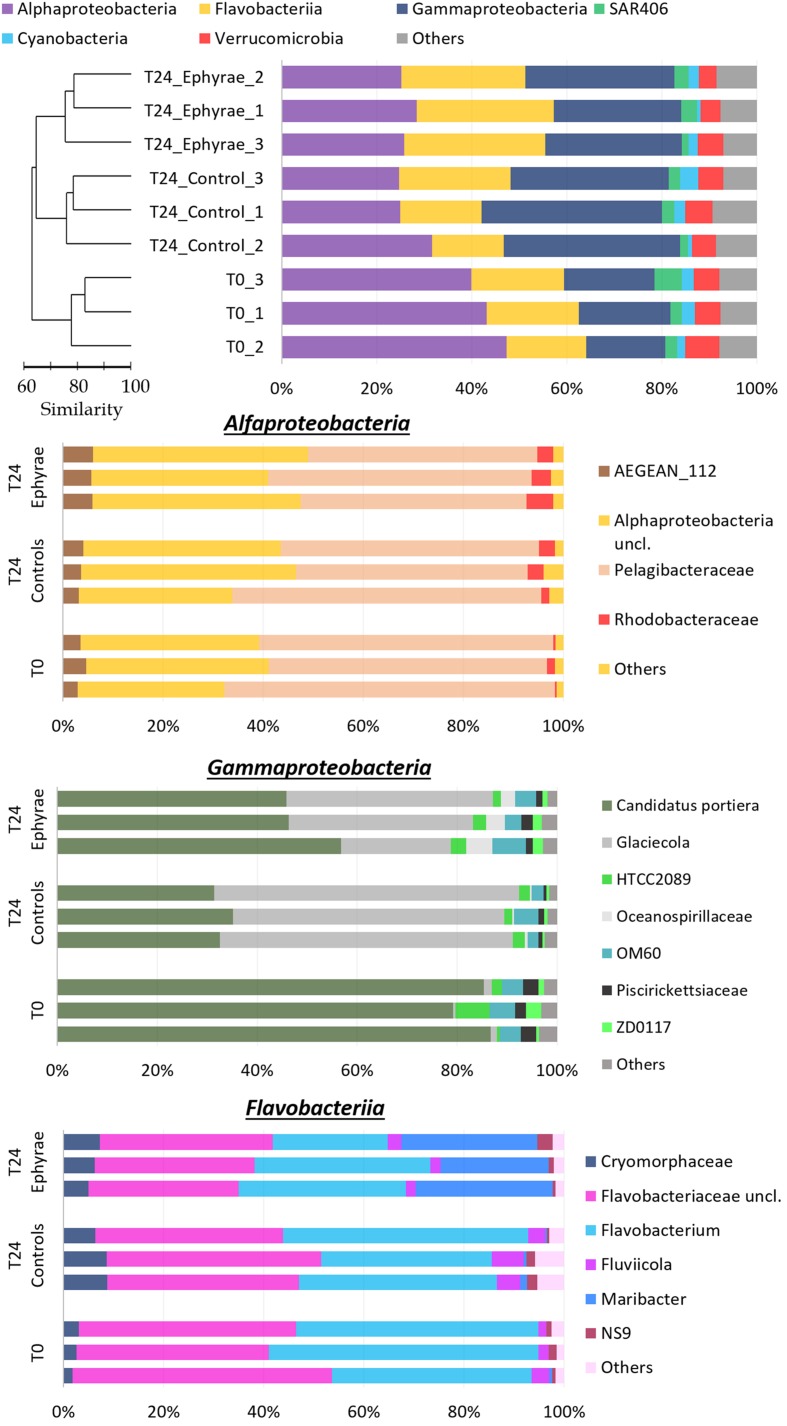
**Profiles of the prokaryotic community composition obtained with the NGS technique.** **(Top)** The most abundant phyla (with RAs > 1%), with classes reported for Proteobacteria and Bacteroidetes. The profiles are ordered according to the cluster analysis shown next to the *Y*-axis. **(Bottom)** Three bar plots showing insights of taxa composition of Alphaproteobacteria, Gammaproteobacteria, and Flavobacteriia. Taxa with RAs < 1% were grouped as “Others.”

The profiles of prokaryotic communities (**Figure 5** and **Supplementary Figure 2**; Top) showed that the most abundant taxa were: for Proteobacteria, the classes Alphaproteobacteria (mean RA, 43.5 ± 3.8%) and Gammaproteobacteria (mean RA, 18.3 ± 1.4%); for Bacteroidetes, the class Flavobacteriia (mean RA, 18.5 ± 1.6%); for phylum Verrucomicrobia (mean RA, 6.0 ± 1.1%); for the uncultured SAR406 (mean RA, 3.5 ± 1.9%); and for Cyanobacteria (mean RA, 2.3 ± 0.6%); Planctomycetes, Actinobacteria and the uncultured SBR1093 were also detected (mean RAs, 1.0, 0.6, 0.3%, respectively). Cyanobacteria included only the genus *Synechococcus*, and sequences classified as chloroplast were grouped together in the category of ‘Others,’ which also included the bulk of the sequences that were unclassified during the BLAST analysis (where the RAs were never >0.1%).

Cluster analysis on the profiles of prokaryotic communities showed three main clusters at 75% similarity: T0, the T24 controls (henceforth referred to as T24_C), and the T24 ephyra treatments (henceforth referred as T24_E), which included their respective replicates; no significant differences were detected among the clusters (ANOSIM, *p* > 0.05). The comparison of the RAs of the most abundant taxa at T0 against T24_C and T24_E showed that the Alphaproteobacteria RA significantly decreased to the similar RAs of 27.1% ± 4.0% in T24_C, and 26.5% ± 1.7% in T24_E (Mann–Whitney test, *p* < 0.05). For T24_E, the Flavobacteriia RA increased to 28.2% ± 1.9%, and the Gammaproteobacteria RA increased to 28.9% ± 2.3% (Mann–Whitney test, both *p* < 0.05), while for T24_C, only the Gammaproteobacteria RA greatly increased to 36.1% ± 2.5% (Mann–Whitney test, *p* < 0.05). The RAs for Verrucomicrobia, SAR406, Cyanobacteria and ‘Others’ showed no significant variations among the clusters (Mann–Whitney test, all *p* > 0.05).

Similar to protists, insight analysis was performed on the taxa composition for Alphaproteobacteria, Gammaproteobacteria and Flavobacteriia (**Figure 5** and **Supplementary Figure 2**; Bottom). *Candidatus* portiera dominated the Gammaproteobacteria group composition, although no significant variations were detected at T24 for both of the controls and the treatments (RAs, 15.3 ± 0.9% at T0, 12.0 ± 1.5% at T24_C, 14.3 ± 2.0% at T24_E; *DESeq* tests, both *p* > 0.05). The increment in Gammaproteobacteria was correlated with the significant increase in RA of the genus *Glaciecola*, from 0.2 ± 0.1% at T0, to 9.7 ± 3.3% at T24_E (*DESeq* test, *p* < 0.001), and 20.9 ± 0.8% at T24_C (*DESeq* test, *p* < 0.001). The Flavobacteriia profiles of T0 and T24_C were similar, while for T24_E, the RA of Maribacter increased greatly (*DESeq* test, *p* < 0.001) from 6.31% to 12.9% ± 1.8%. The large decrease in the Alphaproteobacteria group was correlated mainly with the decrease in the Pelagibacteraceae RA (*DESeq* test, *p* < 0.01 for both T24).

## Discussion

### Predation on Microplankton

The feeding on microplankton organisms by the adult stage of *Aurelia* has already been reported in the literature (Stoecker et al., 1987; Båmstedt, 1990; Uye and Shimauchi, 2005; Malej et al., 2007; Lo and Chen, 2008), although most studies addressed the impact on larger organisms (e.g., Elliott and Leggett, 1997; Hansson et al., 2005; Titelman and Hansson, 2006; Møller and Riisgård, 2007; Riisgård and Madsen, 2011). In the present study, to the best of our knowledge, this microcosm grazing experiment is the first analysis of *A. aurita* ephyrae predation on the aloricate ciliates, tintinnids, micrometazoans, Dinophyceae, Coccolithophyceae, Coscinodiscophyceae, Fragilariophyceae, Dictyochophyceae, Mediophyceae, and Bacillariophyceae communities. We also assessed the predation impact of *A. aurita* ephyrae on both pigmented and heterotrophic nanoflagellates, although we found no evidence of their feeding activity on these organisms. The estimated *A. aurita* ephyrae ingestion rates on microplanktonic taxa were relatively high considering the prey availability, and the five ephyrae that were added in each microcosm led to large reductions in the abundances and biomasses of these communities in just 24 h.

The correlations revealed here between the ephyra relative ingestion rates and the prey relative initial abundances, and the lack of correlation between the ephyra relative ingestion rates and the prey biovolumes, suggest that there was no selection based on prey size. Thus, the ephyrae appeared to prey preferentially upon what was more available. Furthermore, considering the ratios between the ephyra ingestion rates and the prey initial abundances, for the aloricate ciliates, tintinnids, and Dinophyceae taxa, these were on average >1, while for the micrometazoans, Coccolithophyceae, Bacillariophyceae, and Coscinodiscophyceae, these were on average <1. Only the taxa belonging to Mediophyceae, Fragilariophyceae, and the genus *Pseudonitzschia* had ratios comparable with those of the aloricate ciliates, tintinnids, and Dinophyceae, although this was probably because they were in colonies of two organisms (i.e., a single capture event might lead to double ingestion). Although we have no data on the growth rates for these organisms, comparison of their abundances at T0 and T24 in the controls with those at T24 in the ephyra treatments showed greater reduction for the aloricate ciliates, tintinnids and Dinophyceae, where the mean abundance was more than halved.

This suggests that the ephyrae might prey selectively on some groups of organisms over others. This partially contradicts the widespread evidence of *A. aurita* as a generalist feeder, due to its ability to exploit a wide range of marine organisms and the lack of clear evidence for patterns of prey selection (for detailed references, see Introduction); nevertheless, the data in the present study suggest selective ingestion of aloricate ciliates, tintinnids and Dinophyceae for the juvenile stage of *A. aurita.* According to the report of Suchman and Sullivan (2000), a possible explanation for this is the differential vulnerability of the prey, specifically due to the differences in sizes, concentrations, and motility.

Size, cell abundance, and motility positively influence prey–predator encounter rates (Gerritsen and Strickler, 1977, Pastorok, 1981). For size and concentration, clear differences emerged in the present study in the comparison of the biovolumes of the ephyra-ingested taxa with their respective abundances: the Bacillariophyceae and Mediophyceae densities were higher than those of aloricate ciliates, tintinnids, and Dinophyceae, but this latter group included larger organisms than the Bacillariophyceae and Mediophyceae; however, >75% of the ephyra-ingested aloricate ciliates, tintinnids and Dinophyceae were smaller than 20 × 10^3^ μm^3^, and thus in the same size range as the Bacillariophyceae and Mediophyceae. Hence, the prey selected by these *A. aurita* ephyrae was not the most abundant, nor even the largest.

In addition to the encounter rate, the prey motility affects the capture efficiency, in agreement with the theory proposed by Costello and Colin (1994) of marginal flow velocity. However, while this theory applies to mesoplankton and micrometazoans that can swim relative fast and thus have an escape strategy (Sullivan et al., 1994), it does not fit for other microplanktonic organisms that have limited swimming capability. Anyhow, Dinophyceae, aloricate ciliates, and tintinnids are generally characterized by higher motility than Bacillariophyceae, Mediophyceae, and Fragilariophyceae, and prey motility might have a critical role during the contact with the *A. aurita* ephyrae tentacles, and thus on the prey recognition mechanism. Sullivan et al. (1997) demonstrated that copepod nauplii that ‘play dead’ after their entrapment in the *A. aurita* feeding current can minimize their chance of contacting the ephyrae tentacles and thus be expelled from the subumbrella. Regula et al. (2009) hypothesized that only particles recognized as food will trigger the nematocyst, and the chemicals released from the prey after contact with the nematocyst stimulate the feeding behavior of scyphozoans (Arai, 1997). Indeed, the hydromedusa *Aglaura hematoma* that preys on protists does not react to non-motile prey, such as diatoms and dead nauplii (Colin et al., 2005). Hence even small differences in motility (i.e., the almost lack of motility of Bacillariophyceae and Mediophyceae, versus the relatively slow swimming of Dinophyceae, aloricate ciliates, and tintinnids) might produce relevant differences in prey vulnerability and prey selection.

The analysis of protist diversity through the NGS approach allowed the determination in the present study of the modifications in community compositions with sharper resolution than obtained for the microscopy analysis. NGS is not free from flaws though, which can include: mismatches between the morphological concept of species and OTUs that result from DNA segment comparisons (Orsini et al., 2004); low taxonomic accuracy of some taxa, due to limitations in the reference databases (a large number of sequences here that belonged to 221 different OTUs were grouped together as ‘dinophyceae unclassified’); and difficulties in the comparisons of the number of sequences (by NGS) with the number of organisms (under the microscope).

The cluster analysis for the protist OTU table showed that the profiles for the T0 (with the exception of T0_1) and T24 controls were more similar than the profiles of the T24 ephyra treatments. This outcome supports the hypothesis that predation by *A. aurita* ephyrae can affect the protist community composition.

From the analysis of the NGS data, the groups that were most impacted upon (as *A. aurita* ephyrae prey) were Ciliophora and Dinophyta, which showed reduced RAs, while the Stramenopiles RAs increased. This is in agreement with the commented on ingestion rates regarding the selective predation on aloricate ciliates, tintinnids, and Dinophyceae. However, the insight analysis of the family compositions of Dinophyta, Ciliophora, and Stramenopiles revealed that although the presence of the *A. aurita* ephyrae appeared to benefit some families (e.g., *Stenosemellidae*, chrysophyceae–synurophyceae clade-F) and to disadvantaged others (e.g., *Strombilidiidae, Tintinnidae*, Raphid-Pennate), the RAs of the higher taxonomic groups did not show any large modifications of their family-level compositions.

These *A. aurita* ephyrae thus influenced the microplankton community. These small jellyfish appear to profit from different mechanical–physiological features related with their small body size (5 mm) that makes them able to handle small prey (10–200 μm). Nevertheless, this should be more specifically addressed also for the adult stages of *A. aurita*, to provide a more detailed picture of the impact of this jellyfish during its planktonic stages. Furthermore, this ephyrae predation activity more than halved the communities of the typical microplanktonic grazers, with possible critical consequences on the food-web structure that might become even greater in the light of the increasing trends in their abundance and blooms.

### Shaping of Prokaryote Communities

This grazing experiment with *A. aurita* ephyrae has confirmed the influence that these can have on prokaryotic communities. During the 24-h incubations, the heterotrophic fraction showed increased abundance, especially in the ephyra-treated microcosms (in terms of biomass, by up to 45%). On the contrary, the *Synechococcus* abundance showed a small decrease for the ephyra treatments and a small increase at the end of the control incubation, although these differences were not statistically significant. These data were also confirmed by the NGS data (mean RAs, 2.3% at T0, to 2.4% at T24 in controls, and to 1.6% at T24 for ephyra treatment). There were increasing trends within the ephyra treatments also for HCP and the exoenzymatic activity of leucine aminopeptidase, which supports the growth of heterotrophic prokaryotes.

These data are in agreement with those of Turk et al. (2008) and with the field observations of Riemann et al. (2006), although in other studies with longer incubations, higher HCPs were achieved. Tinta et al. (2012) exposed prokaryotes to jellyfish homogenates (12.5 g L^-1^, w/w) and obtained a mean HCP of 11.8 μg C L^-1^ h^-1^ after 3 days of incubation, while Blanchet et al. (2014) provided DOM from jellyfish (which is more bioavailable) and reported HCP > 10 μg C L^-1^ h^-1^ already within the first day.

On the contrary, in the present study, the jellyfish were alive, and in agreement with the review by Pitt et al. (2009), they might have enriched the microcosms in organic matter (and inorganic nutrients) through the production of mucus, feces and excretions; also, their sloppy feeding and egestion of partially digested prey might have an important role, although these effects have not been studied yet. All of these sources of organic matter can be used to sustain prokaryotic growth. It has been reported previously that excretion produces mainly labile or superlabile *N*-rich organic matter (Pitt et al., 2009). On the contrary, very little information is available on the potential utilization of jellyfish-derived mucus. Mucus of *Aurelia* has been shown to have the same biochemical composition as the jellyfish body (Ducklow and Mitchell, 1979), and is produced in large quantities (Heeger and Möller, 1987; Arai, 1997), although it might not be immediately available. Indeed, this matrix can be a complex and heterogeneous source of particulate (e.g., micro-aggregates, macro-aggregates) and DOM (in a colloidal form) that will be slowly degraded by exoenzymes due to its biochemical and structural (three dimensional) properties (Wells, 2002; Pitt et al., 2009; Arnosti, 2014).

Therefore, in the treated microcosms of the present study, the presence of the ephyrae enhanced the polypeptide degradation by sixfold to sevenfold the T0 value, which only doubled in the controls. Moreover, an influence of the hydrolysis rates by the prokaryotic consortia associated with the jellyfish on the overall increase in leucine aminopeptidase activity for the ephyra treatments cannot be excluded, as exceptionally high proteolysis was detected when this metabolic feature was tested on single ephyrae (*ca*. 1 μmol ind^-1^ h^-1^). Surprisingly, at T24, there was slower HCP and higher prokaryotic abundance in the treated microcosms. These data imply a 60% reduction in the specific growth rate (HCP/heterotrophic prokaryote biomass) compared to the controls. As the community growth rate is the overall consequence of several drivers, such as temperature (kept constant here), substrate availability (i.e., quantity, quality) and assemblage structure (Church, 2008), we speculate that shifts in the community structure (see below) and the modified complexity of the organic matter provided by the ephyrae were the main causes of the observed growth rates. As the dissolved organic carbon (DOC) concentration of the control microcosms (848.9 ± 83.7 μM) did not significantly differ compared to the ephyra treatments at T24 (906.7 ± 55.0 μM; DOC concentration at T0 = 297.1 ± 2.5 μM; C. Santinelli, personal communication), the difference in the prokaryotic metabolic response is more likely to arise from the organic matter in the particulate phase provided by the ephyrae.

This reshaping of the prokaryotic community induced by the likely input of the organic carbon released by these living jellyfish is similar to the response that was described by Tinta et al. (2012) and Blanchet et al. (2014) after the input of organic matter derived from dead jellyfish. Tinta et al. (2012) found a complete shift in prokaryote communities, which were finally composed of only the Gammaproteobacteria and Flavobacteriia phyla. This was probably related to the lower numbers of 16S rRNA clone library sequences they analyzed, and it was based on 6 days of incubation. Blanchet et al. (2014) used a pyrosequencing approach, and they reported that after 2 days of incubation the community was dominated by Gammaproteobacteria, although the diversity of prokaryote assemblages were not affected. However, after 9 days, when the more labile fraction of the organic matter had been consumed, the Bacteroidetes (the phylum that includes Flavobacteriia) overwhelmed the communities due to their ability to degrade polymeric compounds of organic matter (Fernández-Gómez et al., 2013).

The profile analysis of prokaryotic communities highlighted that modifications occurred in the natural assemblage after the incubations with the *A. aurita* ephyrae. Gammaproteobacteria and Flavobacteriia showed increased RAs coupled with decreased Alphaproteobacteria RA. The phyla that were favored by the presence of jellyfish are usually found in association with particulate matter (Kirchman, 2002; Simon et al., 2002) and can degrade high-molecular-weight organic compounds (Reichenbach, 1992). On the contrary, Alphaproteobacteria was composed mainly of Pelagibacteraceae (formerly known as SAR11; RAs, 45 to 66%), which are known to thrive oligotrophic conditions (Eiler et al., 2009).

The increase in Gammaproteobacteria was correlated with the increase in *Glaciecola*, which outcompeted *Candidatus* Portiera. Although the genus *Candidatus* Portiera would appear to be an error in the Greengenes database (McDonald and Hugenholtz, 2014), this taxon belongs to the order Oceanospirillales, which can degrade hydrocarbons (Mason et al., 2012; Lamendella et al., 2014). The genus *Glaciecola* groups species that usually associate with algae (Uchida and Nakayama, 1993; Bowman et al., 1998), and are thus likely to exploit their exudates. The increase in Flavobacteriia paralleled the increase in *Maribacter*, which outcompeted *Flavobacterium* and the Flavobacteriaceae unclassified taxa. The *Maribacter* genus is characterized by heterotrophic metabolism (Barbeyron et al., 2008) and can degrade macromolecules (Oh et al., 2011).

The *A. aurita* ephyrae thus influenced the occurrence of the dominant prokaryotic taxa. The inferred alteration to the DOC composition trigged a reshaping of the community that favored clades that are more adapted to and can exploit mucus-like organic matter. As similar modifications to the biodiversity were shown when organic matter obtained directly from jellyfish bodies was used (Tinta et al., 2012; Blanchet et al., 2014), we can hypothesize that the lower HCP is due to the lower availability of easily utilizable DOC from the live ephyrae. Interesting continuations of this study would be to seek more detailed characterizations of the quality and lability of the organic matter excreted by these jellyfish, together with an insight into the prokaryotes–DOC interactions.

## Author Contributions

LZ and MC set up and sampled the experiment. LZ performed the molecular analysis (from DNA extraction to NGS sequencing) and the elaboration of counts at the microscope; MC carried out the functional analysis on bacterial assemblages. SF supervised the research; AP supplied molecular facilities and gave his contribution on NGS-data interpretation. All authors contributed in writing the manuscript.

## Conflict of Interest Statement

The authors declare that the research was conducted in the absence of any commercial or financial relationships that could be construed as a potential conflict of interest.

## Footnotes

1 http://drive5.com/usearchdrive5.com

2 www.R-project.org/
