# Supplementary material for: Aurelia aurita Ephyrae Reshape a Coastal Microbial Community
Source: Front Microbiol. 2016 May 19;7:749. doi: 10.3389/fmicb.2016.00749 (PMC4871886; doi:10.3389/fmicb.2016.00749)
Supplement: Supplementary file 1 [file Table_1.PDF]

# *Supplementary Material*

# *Aurelia aurita* ephyrae reshape a coastal microbial community

Luca Zoccarato*, Mauro Celussi, Alberto Pallavicini and Serena Fonda Umani

***Corresponding author: Luca Zoccarato**
Marine Ecology Laboratory
Department of Life Science
University of Trieste
via Giorgieri, 10
34127 Trieste, Italy
Tel: +39-040-5588832
Email: luca.zoccarato.88@gmail.com

## 1. Supplementary Figures

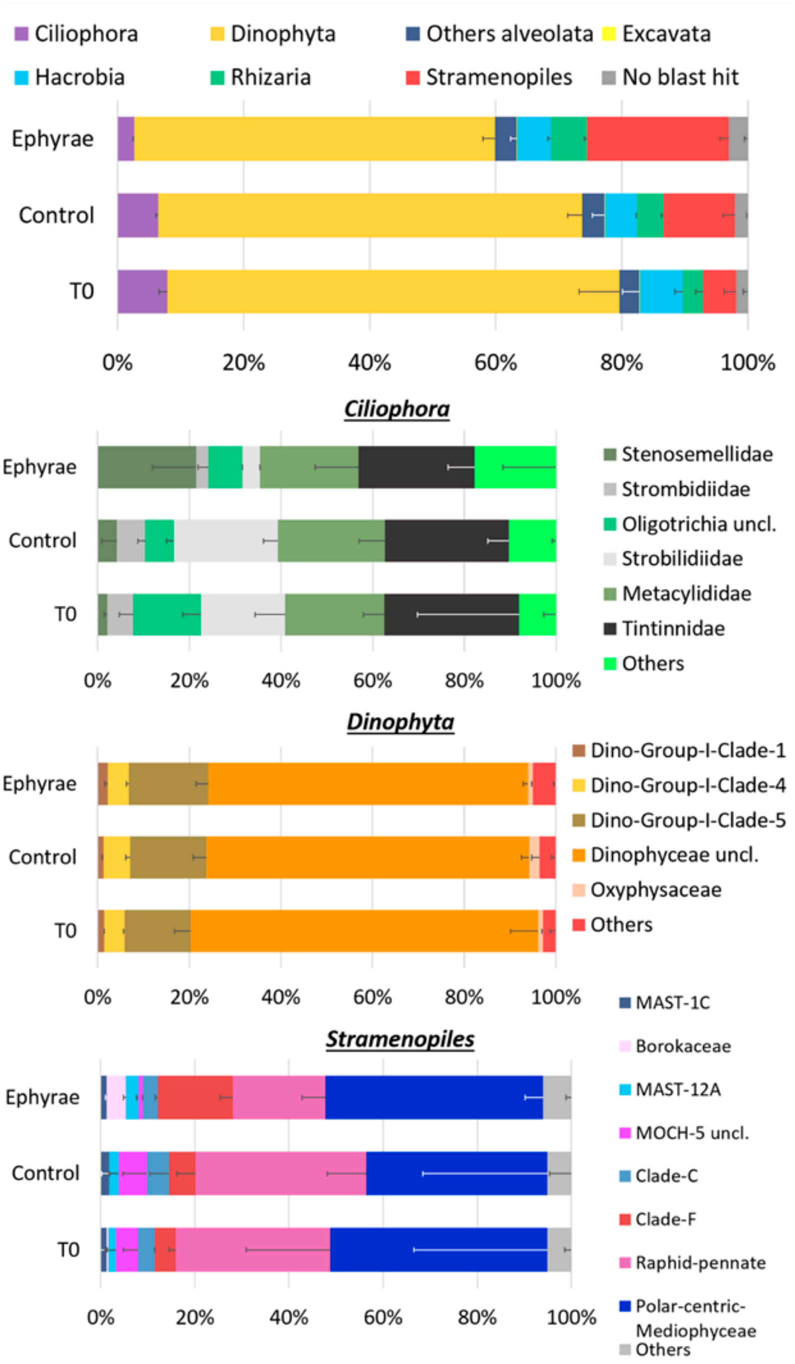


**Supplementary Figure 1.** Profiles of the protist community composition obtained with NGS technique. Top: The major phyla (major division for phylum Alveolata). Bottom: Three bar plots showing the family compositions of Ciliophora, Dinophyta and Stramenopiles. The RA values presented are the average (±SD) of the T0, T24 controls and T24 ephyrae-treatments replicates. Taxa with RAs <1% were grouped as "Others" (RA <5% for Ciliophora).

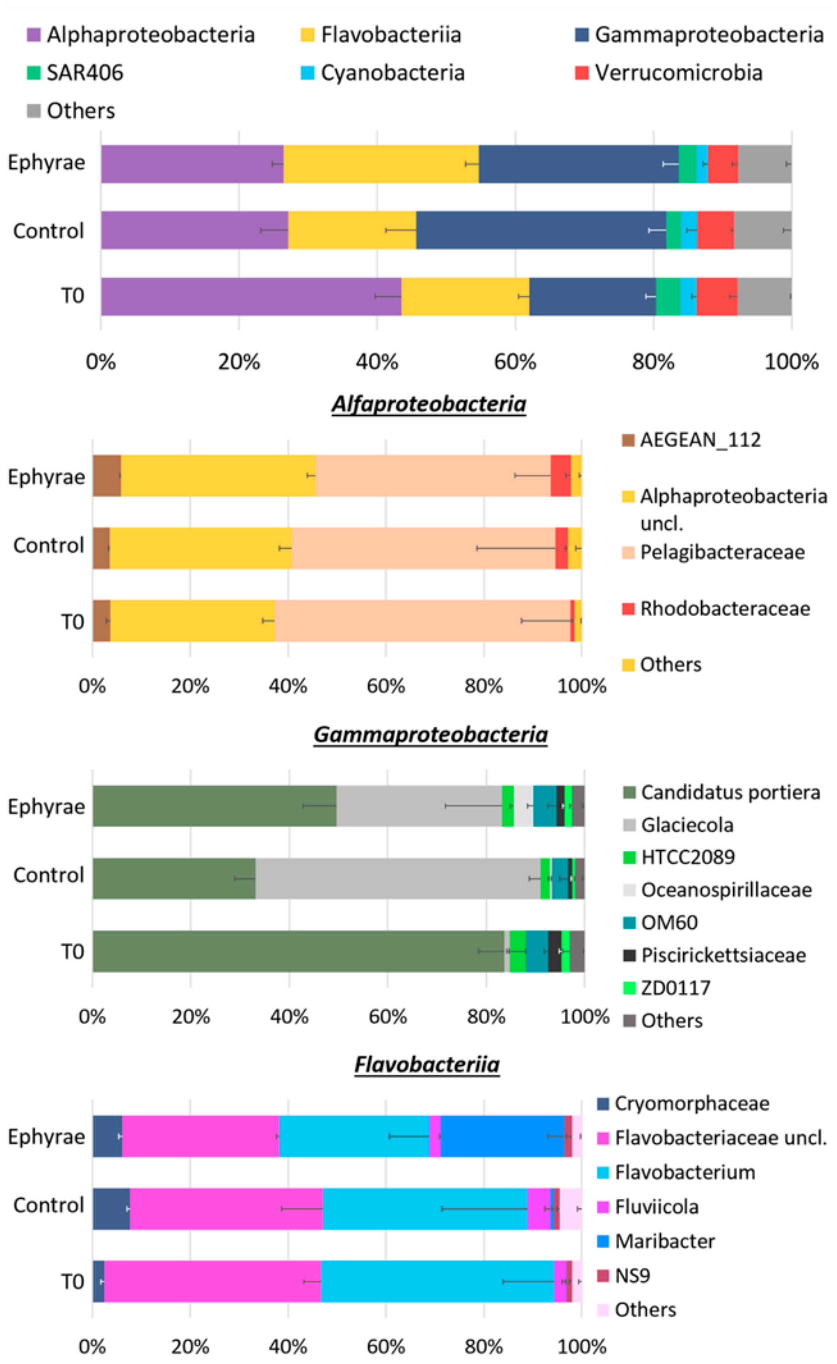


**Supplementary Figure 2.** Profiles of the prokaryotic community composition obtained with NGS technique. Top: The most abundant phyla (with RAs >1%), with classes reported for Proteobacteria and Bacteroidetes. Bottom: Three bar plots showing insights of taxa composition of Alphaproteobacteria, Gammaproteobacteria and Flavobacteriia. The RA values presented are the average (±SD) of the T0, T24 controls and T24 ephyrae-treatments replicates. Taxa with RAs <1% were grouped as "Others".
